# Paradoxical Association Between Baseline Apolipoprotein B and Prognosis in Coronary Artery Disease: A 36,460 Chinese Cohort Study

**DOI:** 10.3389/fcvm.2022.822626

**Published:** 2022-01-25

**Authors:** Huanqiang Li, Bo Wang, Ziling Mai, Sijia Yu, Ziyou Zhou, Hongyu Lu, Wenguang Lai, Qiang Li, Yongquan Yang, Jingru Deng, Ning Tan, Jiyan Chen, Jin Liu, Yong Liu, Shiqun Chen

**Affiliations:** ^1^Department of Cardiology, Guangdong Cardiovascular Institute, Guangdong Provincial People's Hospital, Guangdong Academy of Medical Sciences, Guangzhou, China; ^2^Guangdong Provincial Key Laboratory of Coronary Heart Disease Prevention, Guangdong Cardiovascular Institute, Guangdong Provincial People's Hospital, Guangdong Academy of Medical Sciences, Guangzhou, China; ^3^Guangdong Provincial People's Hospital, School of Biology and Biological Engineering, South China University of Technology, Guangzhou, China; ^4^The Second School of Clinical Medicine, Southern Medical University, Guangzhou, China; ^5^Guangdong Provincial People's Hospital, School of Medicine, South China University of Technology, Guangzhou, China

**Keywords:** apolipoprotein B, coronary artery disease, long-term prognosis, all-cause mortality, paradox

## Abstract

**Background:**

Apolipoprotein B (ApoB) and low-density lipoprotein cholesterol (LDL-C) were identified targets for blood lipid management among coronary artery disease (CAD) patients. However, previous studies reported an inverse correlation between baseline LDL-C concentration and clinical outcomes. This study aims to explore the definite association between baseline ApoB and long-term prognosis.

**Methods:**

A total of 36,460 CAD patients admitted to Guangdong Provincial People's Hospital were enrolled and categorized into two groups: high ApoB (≥65 mg/dL) group and low ApoB (<65 mg/dL) group. The association between baseline ApoB and long-term all-cause mortality was evaluated by the Kaplan-Meier method, Cox regression analyses and restricted cubic splines.

**Results:**

The overall mortality was 12.49% (*n* = 4,554) over a median follow-up period of 5.01 years. Patients with low baseline ApoB levels were paradoxically more likely to get a worse prognosis. There was no obvious difference in risk of long-term all-cause mortality when only adjusted for age, gender, and comorbidity (aHR: 1.07, 95% CI: 0.99–1.16). When CONUT and total bilirubin were adjusted, the risk of long-term all-cause mortality would reduce in the low-ApoB (<65 mg/dL) group (aHR: 0.86, 95% CI: 0.78–0.96). In the fully covariable-adjusted model, patients in the ApoB <65 mg/d group had a 10.00% lower risk of long-term all-cause mortality comparing to patients with ApoB ≥65 mg/dL (aHR: 0.90; 95% CI:0.81–0.99).

**Conclusion:**

This study found a paradoxical association between baseline ApoB and long-term all-cause mortality. Malnutrition and bilirubin mainly mediate the ApoB paradox. Increased ApoB concentration remained linearly associated with an increased risk of long-term all-cause mortality.

## Introduction

It is a well-established association between dyslipidaemias with the occurrence of coronary artery disease (CAD) (1–3) and increased risk of adverse outcomes in CAD patients (4). Additionally, lipid lowing therapy has been proved to benefit CAD patients' prognosis (5–7). For dyslipidemia management, the ESC guideline recommended apolipoprotein B (ApoB) and low-density lipoprotein cholesterol (LDL-C) as the secondary and the primary target, respectively (8, 9). However, several previous studies repeatedly reported that hypercholesterolemia on admission with elevated baseline concentration of LDL-C was paradoxically associated with decreased risk of poor outcomes among CAD patients (10–15).

Moreover, ApoB is the major apolipoprotein and a resonable estimate of the atherogenic lipoprotein families including LDL-C. Usually, patients with higher plasma ApoB will accumulate lipids more and faster, leading to worse prognosis (8). Elevated ApoB can also increase the risk of CAD, even if LDL-C is at an average level (16). Furthermore, a previous study demonstrated that ApoB could better reflect risk of mortality than LDL-C level in patients treated with statin (17). However, few studies reported the association between baseline ApoB level on admission and long-term all-cause mortality.

Therefore, this study aims to elucidate the definite association between baseline ApoB level and risk of long-term all-cause death in patients complicated with CAD.

## Methods

### Study Design and Participants

**Figure 1:**
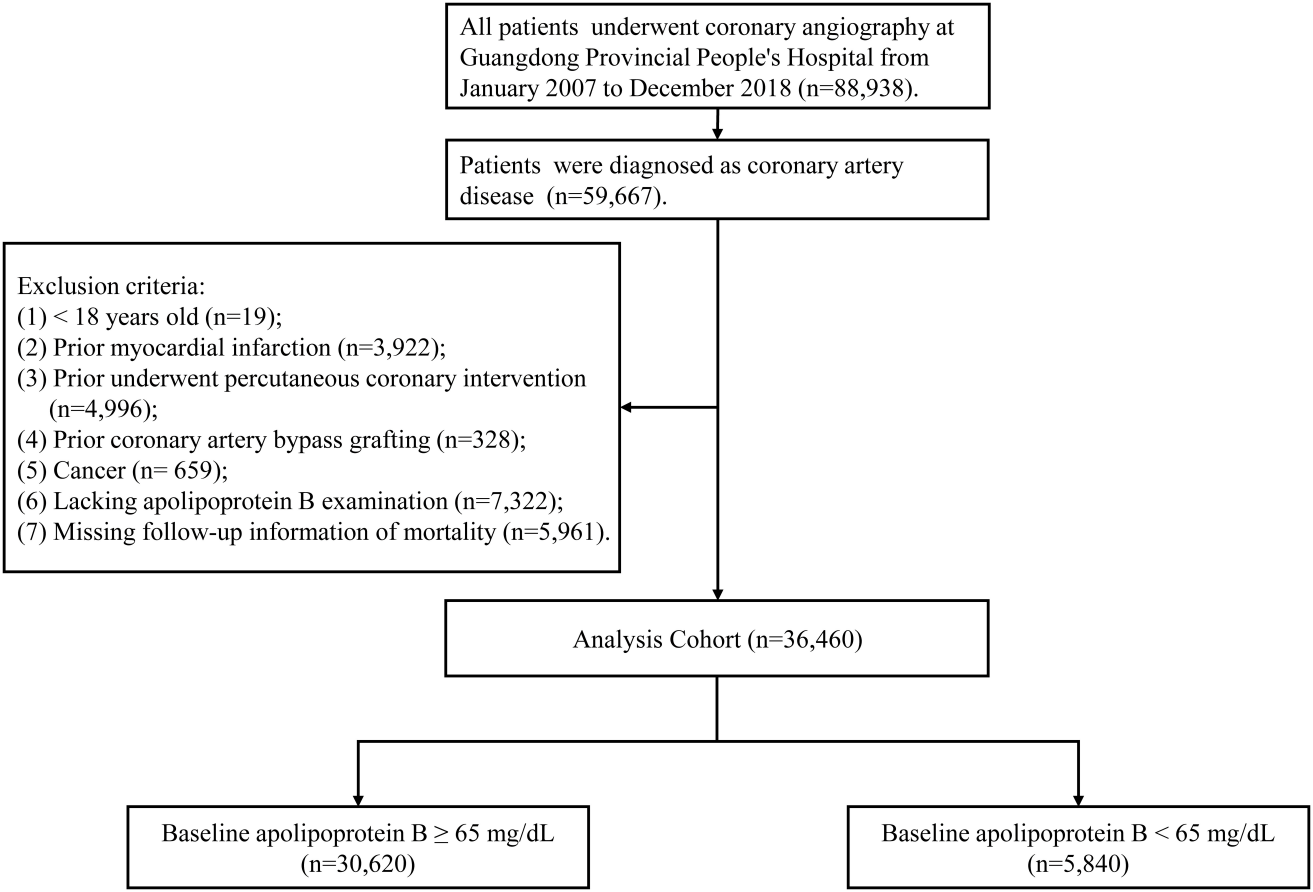
Study flow chart.

Our study cohort stemmed from a previous retrospective cohort of 88,938 patients with coronary angiography (CAG) and percutaneous coronary intervention (PCI) treatment at Guangdong Provincial People's Hospital from January 2007 to December 2018. (Clinicaltrials.gov NCT04407936). The diagnosis of CAD was the basic condition of the patients enrolled (*N* = 59,667) in the current study. Patients who met the following criteria were excluded:under 18 years of age (*n* = 19), previous myocardial infarction (*n* = 3922), pervious underwent percutaneous coronary intervention (*n* = 4,996), previous underwent coronary artery bypass grafting (*n*= 328), cancer (*n* = 659), missing the data of ApoB (n = 7,322), and lacking follow-up data about mortality (*n* = 5,961). Finally, 36,460 CAD patients without the conditions above were enrolled in the study (Figure 1). Guangdong Provincial People's Hospital approved the study proposal [No. GDREC2019555H(R1)]. The study was implemented following the Declaration of Helsinki.

### Procedures

The data of this study came from the electronic medical record system. Baseline nutritional status data, coexistence conditions, demographic characteristics, laboratory examination, and medicine at discharge were collected. Blood samples were taken on admission for hematology and chemistry or collected before PCI/CAG, which obeyed standard clinical practice guidelines (18–20). Follow-up data on patient mortality was obtained and recorded from the Public Security system of Guangdong Province.

### Outcome and Definition

In this study, all-cause mortality during the study period was considered as the primary outcome. CAD was confirmed by CAG (defined as >50% stenosis in at least one vessel) and differentiated in the 10th Revision Codes of the International Classification of Diseases. Additionally, comorbidities consist of several diseases. CHF was defined as Killip class ≥2 or New York Heart Association class ≥3 (21). eGFR <60 ml/min per 1.73 m^2^ was defined as CKD (CKD stage 3 or worse) (22–24). According to the World Health Organization, anemia was defined as hematocrit <39% in males and 36% in females (25). Nutritional status was evaluated using the Controlled nutritional status (CONUT) scoring system. CONUT score is a comprehensive evaluation of serum albumin concentration, the total number of peripheral blood lymphocytes, and total cholesterol concentration. Different scores correspond to different nutritional status (0–1 is normal; 2–4 represents mild malnutrition; >4 for severe malnutrition) (26).

### Statistical Analysis

Referring to the concentration of ApoB, patients were categorized into two groups: a group with a high concentration of ApoB (≥65 mg/dL) and a group with a low concentration of ApoB (<65 mg/dL), following the ApoB goal attainment of 2019 ESC guidelines for dyslipidemia management (8). The categorical variables of descriptive statistics are expressed as quantity (percentage), the continuous variables of the normal distribution are expressed as mean [standard deviation (SD)], and the continuous variables of abnormal distribution are expressed as median [quartile range (IQR)]. Continuous and normally distributed variables were analyzed by independent sample Student *t*-test and Pearson chi-square tests for categorical ones. Kaplan-Meier method and log-rank *t-*test were selected to analyze the difference in survival between two groups. We conducted adjusted and unadjusted Cox proportional risk models to evaluate the link between apolipoprotein cholesterol levels and long-term all-cause mortality. We build a multivariate Cox regression model which incorporates baseline variables clinically relevant. In our study, we selected variables cautiously based on the given number of available events for assurance of the simplicity in the final model. We have successively constructed four models in turn, each of which has or has not been adjusted for concomitant variable: (1) univariate model; (2) adjusted age, gender, and complications including AMI, CHF, hypertension, diabetes mellitus, CKD, anemia, atrial fibrillation, COPD, and stroke; (3) adjusted nutritional status (CONUT) and total bilirubin; (4) adjusted for all covariates. The correlation between ApoB, nutritional status and total bilirubin were analyzed by using spearman analysis. We also constructed the restricted cubic spline curve based on the models above to evaluate the potential non-linear association between baseline ApoB levels and all-cause mortality. Furthermore, we additionally adjusted the use of statin for sensitivity analysis based on model 3 and model 4, respectively. Statistical analyses have been performed by using R software, version 3.6.3. Data would be considered statistically significant with an adjusted *P* < 0.05.

## Results

### Patients' Clinical Characteristics

The final analysis consists of 36,460 CAD patients who met the criteria. Enrolled patients' baseline characteristics have been reported in Table 1. The average ages of patients with ApoB <65 mg/dL and the group of patients with ApoB ≥ 65 mg/dL were 64.50 years and 62.65 years, respectively. Patients with ApoB <65 mg/dL were more likely to develop comorbidities, worse nutrition status, and higher total bilirubin than patients with ApoB ≥65 mg/dL.

**Table 1:** Baseline characteristics.

Characteristic^*^	**Overall (*N* = 36,460)**	**ApoB <65 mg/dL (*N* = 5,840)**	**ApoB ≥65 mg/dL (*N* = 30,620)**	***P* value**
**Demographic characteristics**				
Age, year	62.95 (10.61)	64.50 (10.75)	62.65 (10.56)	<0.001
Age ≥75 years, *n* (%)	5,317 (14.58)	1,107 (18.96)	4,210 (13.75)	<0.001
Male, *n* (%)	27,353 (75.02)	4,557 (78.03)	22,796 (74.45)	<0.001
**Coexisting conditions**				
AMI, *n* (%)	8,022 (22.00)	925 (15.84)	7,097 (23.17)	<0.001
PCI, *n* (%)	26,809 (73.53)	4,071 (69.71)	22,738 (74.26)	<0.001
CHF, *n* (%)	3,434 (9.42)	444 (7.60)	2,990 (9.76)	<0.001
Hypertension, *n* (%)	20,534 (56.32)	3,460 (59.25)	17,074 (55.76)	<0.001
Diabetes mellitus, *n* (%)	9,825 (26.95)	1,708 (29.25)	8,117 (26.51)	<0.001
CKD, *n* (%)	7586 (21.75)	1,295 (23.22)	6,291 (21.47)	0.004
Anemia, *n* (%)	11,262 (31.84)	2,309 (40.90)	8,953 (30.11)	<0.001
Atrial fibrillation, *n* (%)	1,146 (3.14)	219 (3.75)	927 (3.03)	0.004
COPD, *n* (%)	311 (0.85)	57 (0.98)	254 (0.83)	0.298
Stroke, *n* (%)	2,051 (5.63)	382 (6.54)	1,669 (5.45)	0.001
**Nutritional status**				
Without malnutrition, *n* (%)	15,271 (44.19)	561 (10.19)	14,710 (50.64)	<0.001
Mild malnutrition, *n* (%)	15,261 (44.16)	3,739 (67.90)	11,522 (39.66)	<0.001
Moderate malnutrition, *n* (%)	3,812 (11.03)	1,125 (20.43)	2,687 (9.25)	<0.001
Severe malnutrition, *n* (%)	212 (0.61)	82 (1.49)	130 (0.45)	<0.001
**Laboratory examination**				
TBIL, mmol/L	14.54 (6.74)	14.91 (7.43)	14.48 (6.61)	<0.001
HCT	0.40 (0.05)	0.39 (0.05)	0.40 (0.05)	<0.001
Lymphocyte, 10^9^/L	1.94 (0.71)	1.81 (0.68)	1.97 (0.71)	<0.001
Total cholesterol, mmol/L	4.60 (1.21)	3.29 (0.68)	4.85 (1.13)	<0.001
HDL-C, mmol/L	1.00 (0.26)	0.99 (0.28)	1.00 (0.26)	<0.001
LDL-C, mmol/L	2.85 (0.97)	1.74 (0.42)	3.07 (0.90)	<0.001
Triglyceride, mmol/L	1.67 (1.23)	1.33 (1.42)	1.73 (1.18)	<0.001
ApoB, mg/dL	86.87 (23.90)	55.69 (7.20)	92.82 (21.20)	<0.001
ALB, g/L	36.37 (4.23)	36.14 (4.21)	36.41 (4.23)	<0.001
**Medicine at discharge**				
RASi, *n* (%)	17,550 (48.94)	2,675 (46.94)	14,875 (49.32)	0.001
β-blocker, *n* (%)	28,848 (80.45)	4,479 (78.59)	24,369 (80.80)	<0.001
Statins, *n* (%)	33,921 (94.57)	5,319 (93.33)	28,602 (94.81)	<0.001
**Events**				
All-cause mortality, *n* (%)	4,554 (12.49)	903 (15.46)	3,650 (11.92)	<0.001

* *Data are presented as the mean value (standard deviation) or number of participants (percentage)*.

*ApoB, apolipoprotein B; AMI, acute myocardial infarction; PCI, percutaneous coronary intervention; CHF, congestive heart failure; CKD, chronic kidney disease; COPD, chronic obstructive pulmonary disease; TBIL, total bilirubin; DBIL, direct bilirubin; HDL-C, high-density lipoprotein cholesterol; LDL-C, low-density lipoprotein cholesterol; RASi, renin angiotensin system inhibitor*.

### Main Outcomes

**Figure 2:**
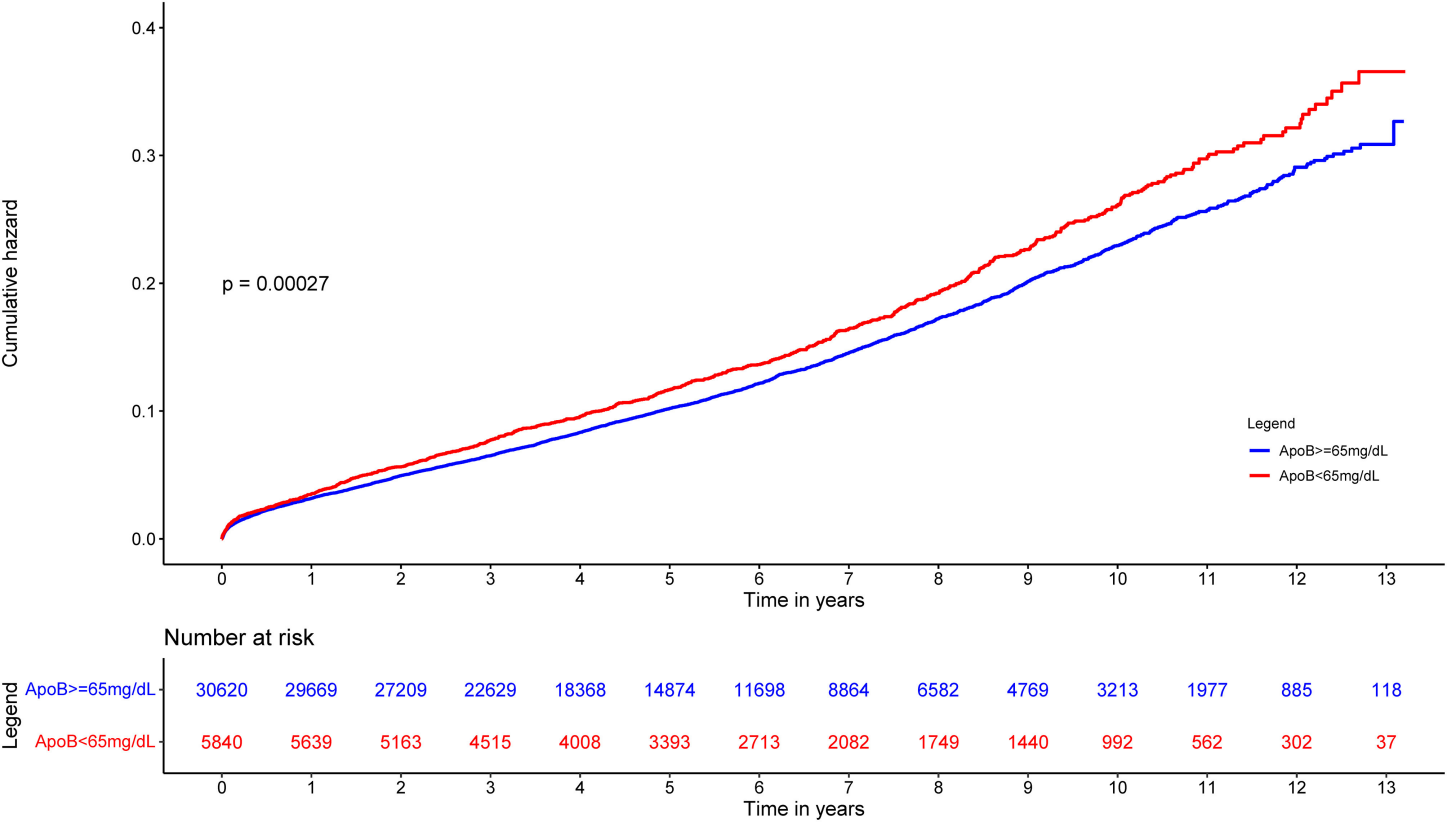
Cumulative incidence of all-cause death for ApoB <65 mg/dL group vs. ApoB ≥65 mg/dL group in CAD patients.

During the whole follow-up period with a median time of 5.01 years (IQR 2.96–7.65), The all-cause mortality rate of the patients surveyed was 12.49% (*n* = 4,553). Kaplan-Meier analysis indicated the phenomenon that patients with low ApoB (<65 mg/dL) had a worse prognosis (Figure 2).

**Figure 3:**
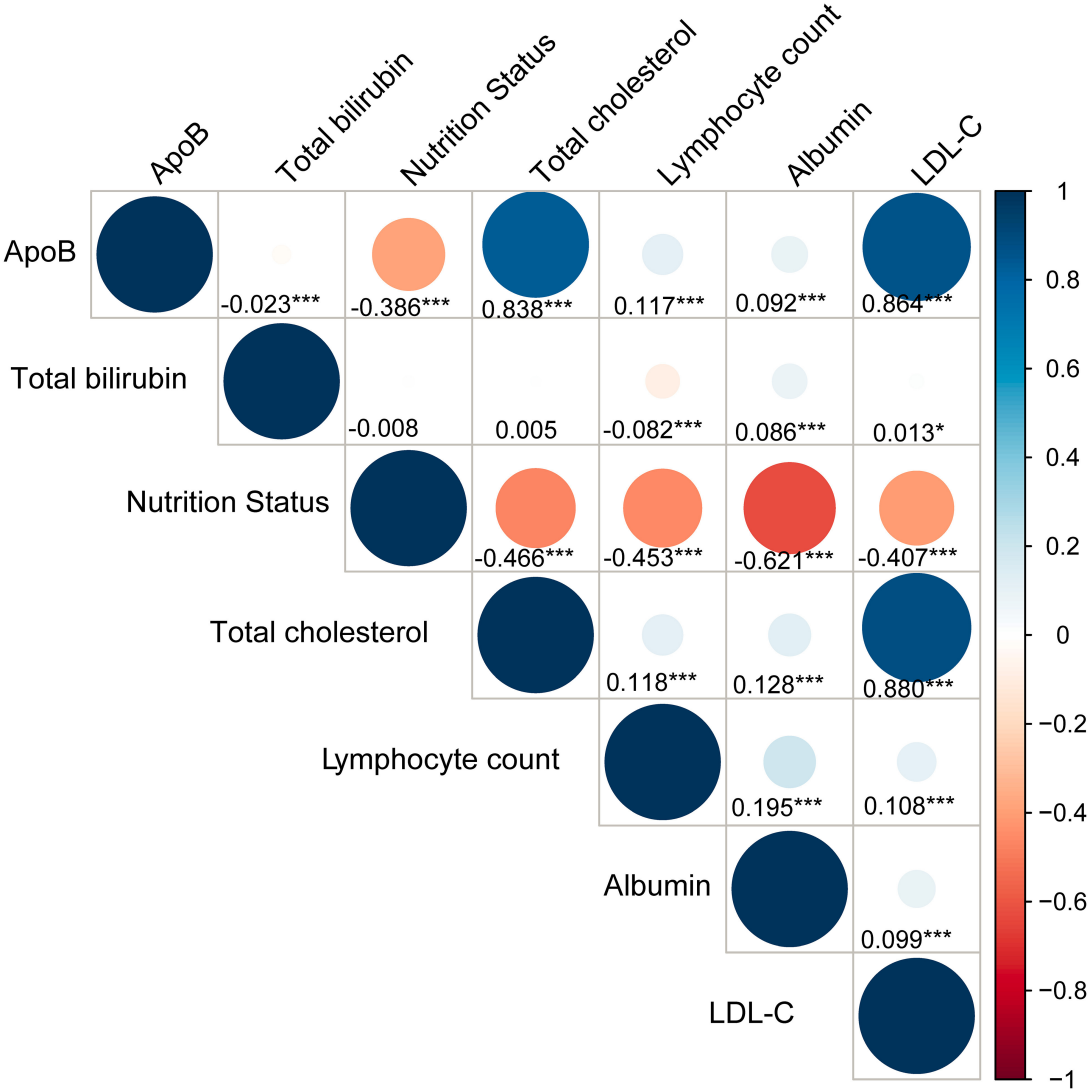
Correlations between ApoB, total bilirubin, nutritional status and its components. Nutritional status is assessed by Controlling Nutritional Status (CONUT) score. Total cholesterol, lymphocyte count and albumin are components of CONUT score. *p* < 0.05 *; *p* < 0.01**; *p* < 0.001***.

The correlations between ApoB and total bilirubin, nutritional status evaluated by CONUT score and components of CONUT score were shown in (Figure 3). ApoB did not show a significant correlation with total bilirubin (r = −0.023, *p* < 0.001) and no strong correlation between ApoB and nutritional status which was evaluated by CONUT score (r = −0.38, *p* < 0.001).

**Figure 4:**
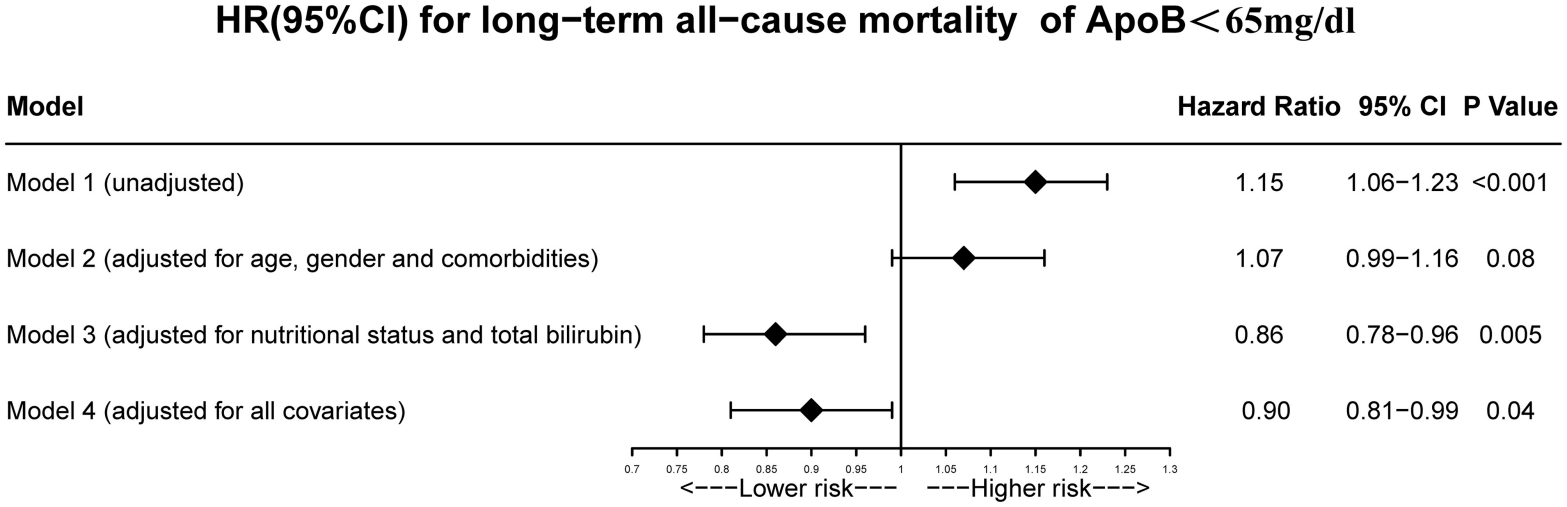
Unadjusted and adjusted HRs and 95% CIs for the primary end point (long-term all-cause mortality) of ApoB <65 mg/dL group vs. ApoB ≥65 mg/dL group in CAD patients. Model 1: Unadjusted model. Model 2: Adjusted for age ≥75 years, sex, PCI and comorbidities including AMI, CHF, hypertension, diabetes mellitus, CKD, anemia, atrial fibrillation, COPD and stroke. Model 3: Adjusted for malnutrition. Model 4: Adjusted for all covariates: age ≥75 years, sex, PCI and comorbidities including AMI, CHF, hypertension, diabetes mellitus, CKD, anemia, atrial fibrillation, COPD, stroke and malnutrition.

Univariate Cox regression model shows that low baseline concentration of ApoB (<65 mg/dL) is associated with a high risk of long-term all-cause mortality in CAD patients. (HR: 1.15, 95% CI: 1.06–1.23, Figure 4) Multivariate Cox regression analyses were then performed to adjust for confounding factors between patients with low ApoB (<65 mg/dL) and patients with high ApoB levels (≥65 mg/dL) (Figure 4). After adjusting for age, gender, and comorbidity (model 2), there was no obvious difference in long-term all-cause mortality between patients with low ApoB (<65 mg/dL) and patients with high ApoB levels (≥65 mg/dL) (aHR: 1.07, 95% CI: 0.99–1.16, Figure 4). Nevertheless, when CONUT and total bilirubin (model 3) were adjusted, the risk of long-term all-cause mortality would reduce for the patients with low-ApoB (<65 mg/dL) (aHR: 0.86, 95% CI: 0.78–0.96, Figure 4). In the fully covariable-adjusted model (model 4), patients with the baseline ApoB <65 mg/dL had a 10.00% lower risk of long-term all-cause death when comparing to patients with ApoB ≥65 mg/dL (aHR: 0.90; 95% CI:0.81–0.99, Figure 4).

**Figure 5:**
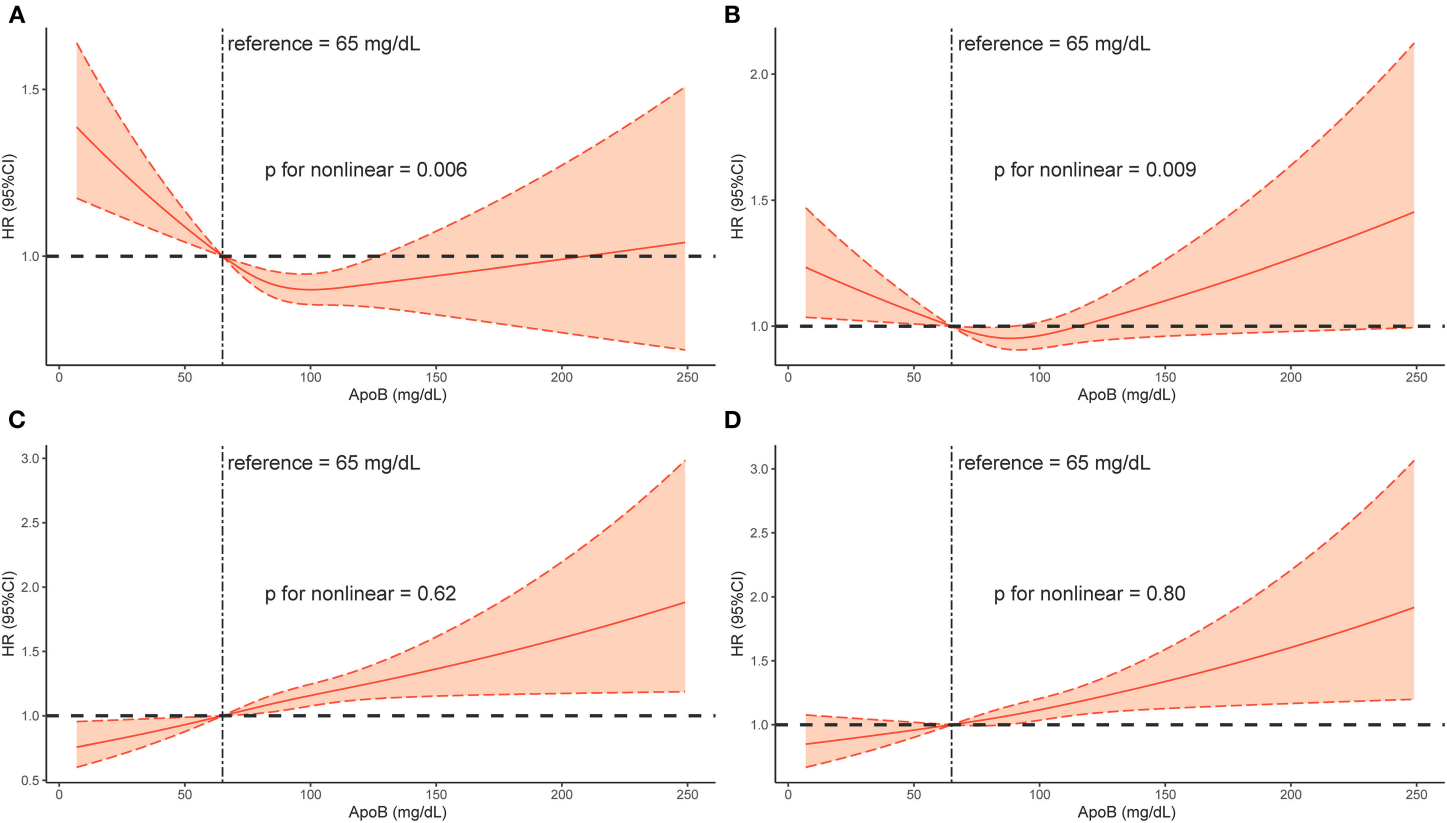
Restricted spline curve of the Baseline ApoB hazard ratio for mortality (long-term all-cause mortality) in CAD patients. **(A)**: Model 1, Unadjusted model. **(B)**: Model 2, Adjusted for age ≥75 years, sex, PCI and comorbidities including AMI, CHF, hypertension, diabetes mellitus, CKD, anemia, atrial fibrillation, COPD and stroke. **(C)**: Model 3, Adjusted for malnutrition. **(D)**: Model 4, Adjusted for all covariates: age ≥75 years, sex, PCI and comorbidities including AMI, CHF, hypertension, diabetes mellitus, CKD, anemia, atrial fibrillation, COPD, stroke and malnutrition.

According to the results of restricted cubic splines, a J-shaped association existed in the univariable model (p for non-linearity = 0.006, Figure 5A) and a U-shaped association existed in the multivariable model adjusting age, gender and comorbidities (p for non-linearity = 0.009, Figure 5B). When CONUT and total bilirubin were adjusted, a linear association existed between baseline ApoB and the risk of long-term all-cause mortality (p for non-linearity = 0.62, Figure 5C; p for non-linearity = 0.80, Figure 5D).

### Sensitivity Analysis

After additional adjustment for the use of statin, the results of Cox regression and restricted cubic splines did not change significantly (Supplementary Figures 1, 2).

## Discussion

To the best of our knowledge, no previous study has demonstrated the relationship between long-term all-cause mortality and baseline serum ApoB concentration in CAD patients. According to the Kaplan-Meier curve, there is a paradoxical association between baseline ApoB concentration and long-term prognosis, in which low baseline ApoB was associated with a high risk of poor outcomes. After considering the baseline discrepancies including age, sex, and comorbidities, the association between low ApoB level and worse prognosis was insignificant. Taking nutrition status and total bilirubin level into consideration, high baseline serum concentration of ApoB was one of the factors which can affect long-term all-cause mortality independently. The apolipoprotein paradox has been observed in CAD patients, but it would no longer exist by taking the effects of malnutrition and bilirubin into account. The paradoxical association between ApoB and worse prognosis was mainly mediated by the effect of bilirubin and underlying malnutrition.

According to our findings, baseline plasma apolipoprotein B levels were negatively associated with long-term prognosis in unadjusted analysis. Although no previous studies have reported similar phenomena, previous studies have reported paradoxical relations between baseline LDLC level and long-term prognosis (10–14). Additionally, ApoB is highly correlated with LDLC (27). These previous studies illustrated that baseline confounders caused the cholesterol paradox of LDL-C. Cho et al. found AMI patients owing lower LDL-C levels (<1.8 mmol/L) on admission would be prone to have higher mortality in crude short-term (10). Meanwhile, patients in the lower LDL-C group were older and had a higher proportion of comorbidities. After adjusting for covariates, the level of LDL-C was not correlated strongly to short-term mortality. Another study conducted by Wang et al. showed that ACS patients' low baseline LDL-C concentration will increase the risk of mortality among hospitalized patients (10). Similarly, after adjustment for baseline confounders, the paradoxical association disappeared, and no apparent relationship between LDL-C levels and in-hospital mortality. The other three trials showed that the reduction of baseline LDL-C concentration was correlated to the increase of mortality in unadjusted and adjusted models (12–14).

All of these studies have found a paradoxical relationship between hyperlipidemia and well-prognosis, but these studies have not found causes for the phenomenon. A study conducted by Wallner et al. indicated that lipid profile would be altered in hyperbilirubinemia patients, characterized by a lower ApoB and LDL-C (28). Previous studies also demonstrated that the bilirubin concentration was negatively associated with LDL-C level (29, 30), and ApoB was the primary structural protein of low-density lipoprotein (LDL) (31). Moreover, increased bilirubin level was associated with an increased risk of poor prognosis (32–34). An observational study retrospectively recruited 3013 patients with AMI conducted by Huang et al. suggesting a positive correlation between serum total bilirubin concentration and short-term mortality among AMI patients (32). In another observational study including 1,167 patients with STEMI who underwent PCI, Baumann et al. found that high levels of total bilirubin (≥12 mg/L) were associated with a 128% increased risk of major adverse cardiac events (MACEs) during hospitalization (33). In addition, Wu et al. demonstrated that for patients with severe systolic heart failure, patients with higher bilirubin levels had conspicuously higher all-cause mortality (34). Since there was a tight connection between ApoB and total cholesterol, which is an evaluation index for nutritional status (26, 27), low ApoB may also indicate potential malnutrition. The CONUT score is an effective and objective tool to assess patients' nutritional status in hospital (26, 35). Roubín et al., Wada et al., and Chen et al. found that the nutritional status obtained by CONUT score can be considered one of the indicators to predict the long-term clinical outcomes of CAD patients (35–37).

Several reasons may explain the result of the present study. Firstly, according to the baseline, patients with low levels of ApoB (<65 mg/dL) were more elderly and complicated with more comorbidities. These confounders were highly associated with poor prognosis. Compared with patients having high levels of ApoB (≥65 mg/dL), patients with low ApoB (<65 mg/dL) had higher prevalence of elderly patients (18.96 vs. 13.75%), hypertension (59.25 vs. 55.76), CKD (23.22 vs. 21.47%), diabetes mellitus (29.25 vs. 26.51%) and anemia (40.90 vs. 30.11%). Thus, our study constructed model 2 in Cox regression which adjusted for age, male, and comorbidities. The results showed baseline ApoB concentration was not relevant to long-term all-cause mortality when taking age, male, and comorbidities into account. Secondly, patients with ApoB <65 mg/dL had higher bilirubin which was associated with worse prognosis. Bilirubin, the ultimate breakdown of hemoglobin, is an endogenous antioxidant with anti-inflammatory properties (38). On the one hand, as a strong antioxidant, bilirubin can inhibit the generation of atherogenic lipid such as oxidized-LDL. It may directly affect lipid metabolism through several pathways, including hepatic very-low-density lipoprotein (VLDL) assembly and cholesterol synthesis, bile cholesterol excretion, and intestinal cholesterol transport (39, 40). Meanwhile, ApoB is a direct measure of circulating numbers of atherogenic lipoproteins and is the main apolipoprotein of cholesterol within LDL, VLDL, and intermediate-density lipoprotein (IDL) particles (27, 41, 42). Thus, it is not surprising that bilirubin was negatively associated with ApoB. On the other hand, serum bilirubin concentration was a sign of heme oxygenase 1 (HO-1) enzyme activity which could be induced by stress, including as part of the acute phase response and complicated artery condition (43–45). Previous studies found that increased levels of serum bilirubin were associated with increased risk of adverse clinical outcomes among patients complicated with cardiovascular disease (46–48). Thirdly, patients with ApoB <65 mg/dL were at poorer nutritional status. In the present study, the prevalence of mild, moderate, severe malnutrition in the low ApoB group (<65 mg/dL) was 67.90, 20.43, and 1.49%, respectively. While, it was 39.66, 9.25, and 0.45% in high ApoB group (≥65 mg/dL), respectively. Evidence is emerging that malnutrition also often represents secondary immune dysfunction (49). Unrecognized malnutrition may cause susceptibility to infection and increase morbidity and mortality. Based on the above literatures, malnutrition and increased bilirubin level were highly associated with increased risk of poor long-term outcomes. The result of model 3 (adjusted for nutrition state, bilirubin) and model 4 (adjusted for all covariates including nutrition state, bilirubin) illustrated a normal association between baseline ApoB concentration and long-term all-cause death.

Lipid management is one of the critical points of secondary prevention in patients with CAD. Well-controlled lipid levels can significantly improve the long-term prognosis of CAD patients. ApoB as the secondary target should be paid attention to risk stratification. All these findings strongly support that physicians also need to incorporate malnutrition identification and bilirubin examination into their daily practice.

### Limitation

Several limitations should be considered. Firstly, the data of the present study was a single-center observational study from China. However, the data of the present study was from a large real-world cohort. Secondly, the data of the included patients were limited, and information such as body weight, BMI, waist circumference, and obesity that might be helpful to evaluate the nutritional status of the patients was missing comprehensively. To compensate for this, we chose the CONUT score based on laboratory examination as a tool to assess nutritional status, which may also help us perform an objective appraisal on patients about their nutritional status. Thirdly, this study only enrolled the ApoB values collected from the patients on admission, making it tougher to evaluate the effect of changes in ApoB levels on clinical endpoints during follow-up. In summary, our study mainly focuses on the clinical importance of baseline ApoB level on prognosis among CAD patients.

## Conclusion

Among CAD patients, there is a paradoxical association between baseline ApoB concentration on admission and the risk of all-cause mortality. Nutrition state and bilirubin levels mediate this abnormal association. Conversely, after considering these two factors, evaluated ApoB concentration remains associated with a worse prognosis.

## Data Availability Statement

The original contributions presented in the study are included in the article/Supplementary Materials, further inquiries can be directed to the corresponding authors.

## Ethics Statement

The studies involving human participants were reviewed and approved by Research Ethics Committee of Guangdong Provincial People's Hospital, Guangdong Academy of Medical Sciences (No. GDREC2019555H[R1]). Written informed consent for participation was not required for this study in accordance with the national legislation and the institutional requirements.

## Author Contributions

HLi, BW, and SC: substantial contributions to the conception and design of the study. ZM, SY, ZZ, WL, QL, and HLu: data collection. SC, YL, JC, YY, JD, JL, and NT: data analysis and/or interpretation of data for the work. HLi, BW, ZM, SY, SC, QL, YY, JD, JL, YL, and JC: drafting of the work or revising it critically for important intellectual content. All authors approved the final version to the manuscript published.

## Funding

This study was supported by the National Natural Science Foundation of China (Grant Nos. 81670339 and 81970311), Beijing Lisheng Cardiovascular Health Foundation (LHJJ20141751), National Key Research and Development Program of China (Grant No. 2016YFC1301202), and Guangdong Provincial People's Hospital Dengfeng Project Fund (DFJH201919 and DFJH2020026).

## Conflict of Interest

The authors declare that the research was conducted in the absence of any commercial or financial relationships that could be construed as a potential conflict of interest.

## Publisher's Note

All claims expressed in this article are solely those of the authors and do not necessarily represent those of their affiliated organizations, or those of the publisher, the editors and the reviewers. Any product that may be evaluated in this article, or claim that may be made by its manufacturer, is not guaranteed or endorsed by the publisher.

## Supplementary Material

Supplementary Figure 1 Unadjusted and adjusted HRs and 95% CIs for the primary end point (long-term all-cause mortality) of ApoB <65 mg/dL group vs. ApoB ≥65 mg/dL group in CAD patients. Model 1: Unadjusted model. Model 2: Adjusted for age ≥75 years, sex, PCI and comorbidities including AMI, CHF, hypertension, diabetes mellitus, CKD, anemia, atrial fibrillation, COPD, stroke and the use of stain. Model 3: Adjusted for malnutrition. Model 4: Adjusted for all covariates: age ≥75 years, sex, PCI and comorbidities including AMI, CHF, hypertension, diabetes mellitus, CKD, anemia, atrial fibrillation, COPD, stroke, malnutrition and the use of stain.

Supplementary Figure 2 Restricted spline curve of the Baseline ApoB hazard ratio for mortality (long-term all-cause mortality) in CAD patients. **(A)**: Model 1, Unadjusted model. **(B)**: Model 2, Adjusted for age ≥75 years, sex, PCI and comorbidities including AMI, CHF, hypertension, diabetes mellitus, CKD, anemia, atrial fibrillation, COPD, stroke and the use of stain. **(C)**: Model 3, Adjusted for malnutrition. **(D)**: Model 4, Adjusted for all covariates: age ≥ 75 years, sex, PCI and comorbidities including AMI, CHF, hypertension, diabetes mellitus, CKD, anemia, atrial fibrillation, COPD, stroke, malnutrition and the use of stain.
