# Supplementary figures and images for: Paradoxical Association Between Baseline Apolipoprotein B and Prognosis in Coronary Artery Disease: A 36,460 Chinese Cohort Study
Source: Front Cardiovasc Med. 2022 Jan 25;9:822626. doi: 10.3389/fcvm.2022.822626 (PMC8821163; doi:10.3389/fcvm.2022.822626)

## HR(95%CI) for long-term all-cause mortality

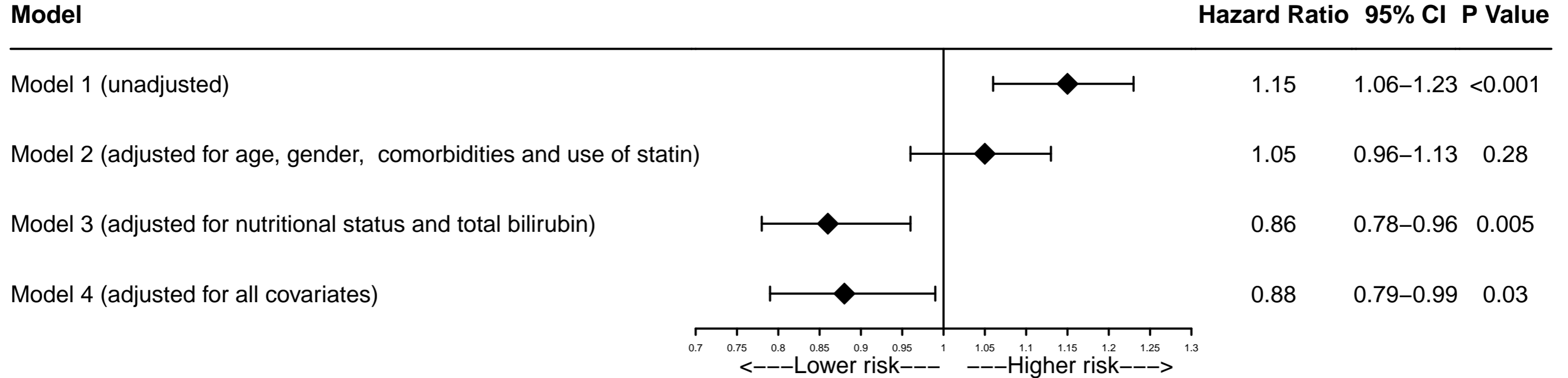

Supplement: Supplementary Figure 1 — Unadjusted and adjusted HRs and 95% CIs for the primary end point (long-term all-cause mortality) of ApoB <65 mg/dL group vs. ApoB ≥65 mg/dL group in CAD patients. Model 1: Unadjusted model. Model 2: Adjusted for age ≥75 years, sex, PCI and comorbidities including AMI, CHF, hypertension, diabetes mellitus, CKD, anemia, atrial fibrillation, COPD, stroke and the use of stain. Model 3: Adjusted for malnutrition. Model 4: Adjusted for all covariates: age ≥75 years, sex, PCI and comorbidities including AMI, CHF, hypertension, diabetes mellitus, CKD, anemia, atrial fibrillation, COPD, stroke, malnutrition and the use of stain. [file Presentation_1.PDF]

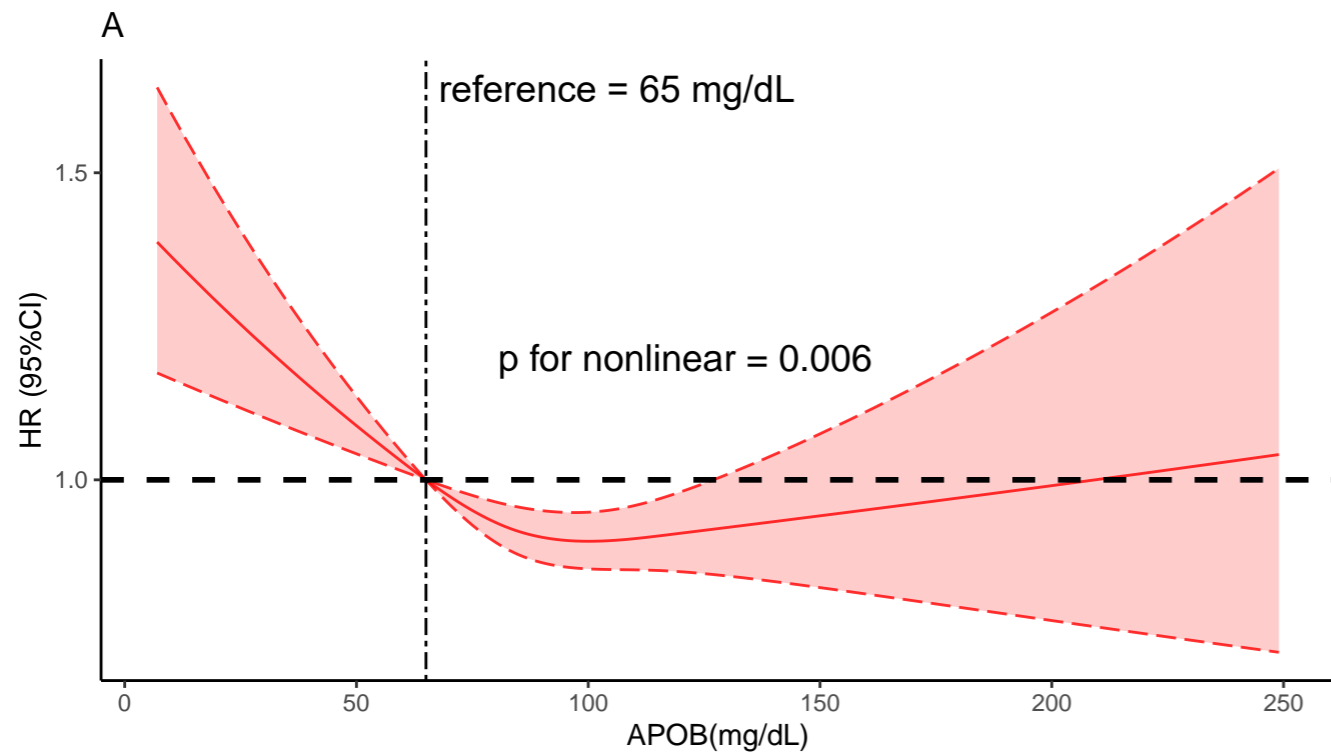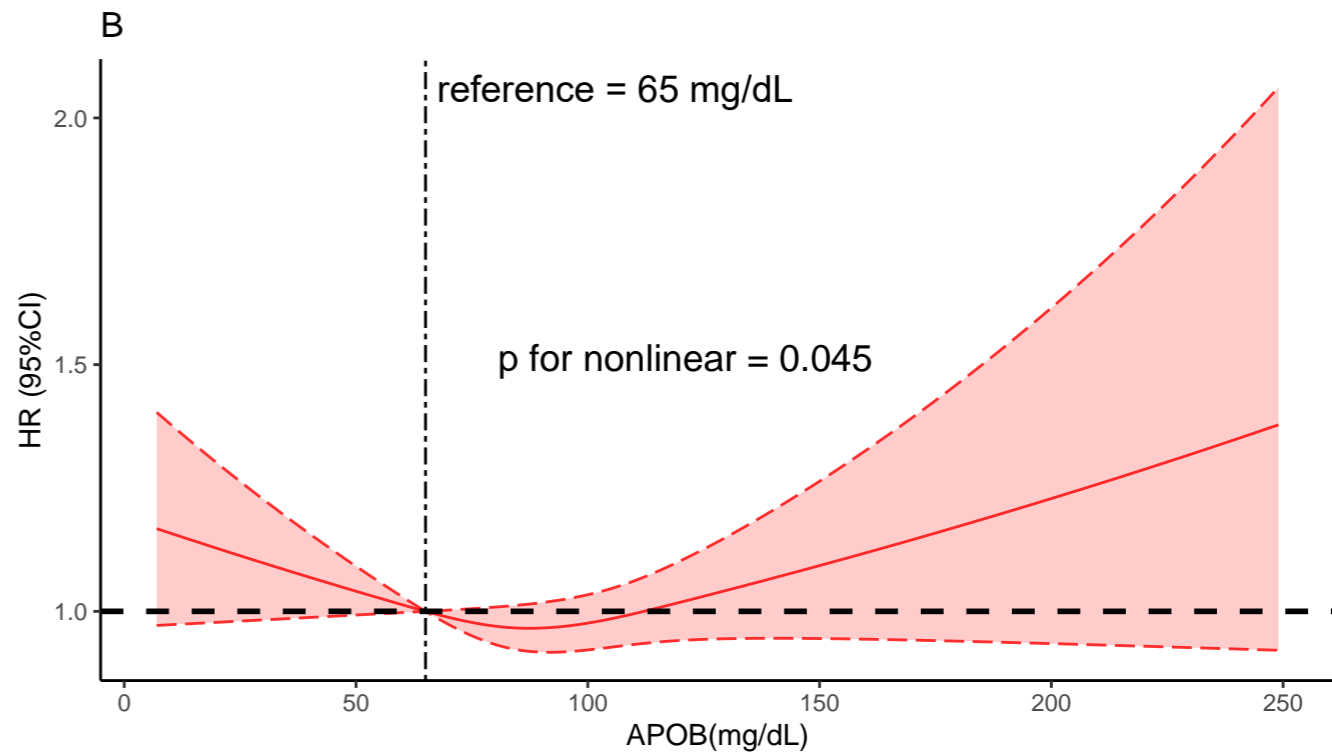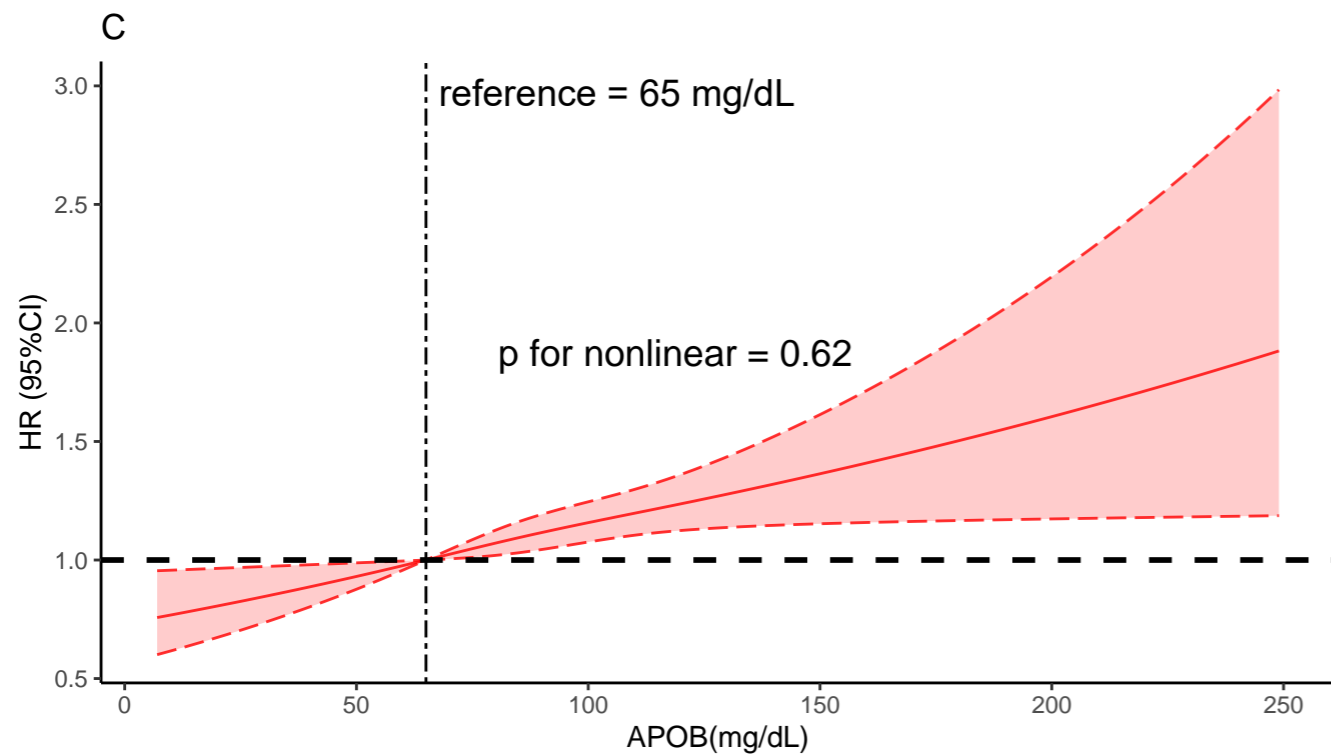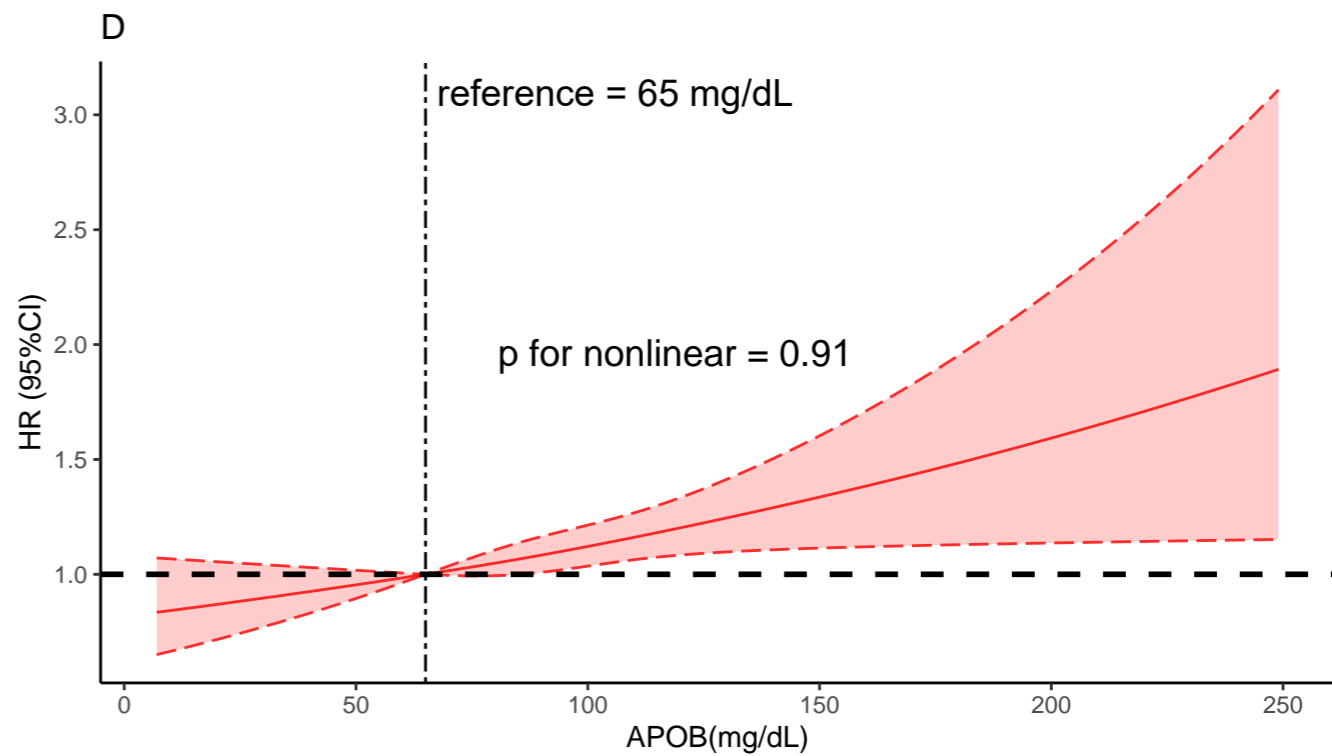

Supplement: Supplementary Figure 2 — Restricted spline curve of the Baseline ApoB hazard ratio for mortality (long-term all-cause mortality) in CAD patients. (A): Model 1, Unadjusted model. (B): Model 2, Adjusted for age ≥75 years, sex, PCI and comorbidities including AMI, CHF, hypertension, diabetes mellitus, CKD, anemia, atrial fibrillation, COPD, stroke and the use of stain. (C): Model 3, Adjusted for malnutrition. (D): Model 4, Adjusted for all covariates: age ≥ 75 years, sex, PCI and comorbidities including AMI, CHF, hypertension, diabetes mellitus, CKD, anemia, atrial fibrillation, COPD, stroke, malnutrition and the use of stain. [file Presentation_2.PDF]
